# Automatic detection of circulating tumor cells in darkfield microscopic images of unstained blood using boosting techniques

**DOI:** 10.1371/journal.pone.0208385

**Published:** 2018-12-13

**Authors:** Anca Ciurte, Cristina Selicean, Olga Soritau, Rares Buiga

**Affiliations:** 1 Department of Computer Science, Technical University of Cluj Napoca, Cluj-Napoca, Romania; 2 Hematology Laboratory, The Oncology Institute “Prof. Dr. Ion Chiricuta”, Cluj-Napoca, Romania; 3 The Oncology Institute “Prof. Dr. Ion Chiricuta”, Cluj-Napoca, Romania; 4 “Iuliu Haţieganu” University of Medicine and Pharmacy, Cluj-Napoca, Cluj-Napoca, Romania; Hunter College, UNITED STATES

## Abstract

Circulating tumor cells (CTCs) are nowadays one of the most promising tumor biomarkers. It is well correlated with overall survival and progression-free survival in breast cancer, as well as in many other types of human cancer. In addition, enumeration and analysis of CTCs could be important for monitoring the response to different therapeutic agents, thus guiding the treatment of cancer patients and offering the promise of a more personalized approach. In this article, we present a new method that could be used for the automatic detection of CTC in blood, based on the microscopic appearance of unstained cells. The proposed method is based on the evaluation of image characteristics and boosting techniques. A dataset of 263 dark field microscopy images was constructed and used for our tests, containing blood spiked with three different types of tumor cells. An overall sensitivity of 92.87% and a specificity of 99.98% were obtained for the detection of CTC, performances which proved to be comparable to those obtained by human experts.

## Introduction

Circulating tumor cells are migratory cells in the blood that allow malignant tumors to establish metastatic colonies distributed throughout the body. These metastases eventually cause the death of the patient. In oncological practice, isolation and analysis of (CTC) could be of great importance, both as a prognostic marker and for continuous monitoring of the response to treatment. CTC identification techniques illustrates the concept of “liquid biopsy” which, unlike conventional biopsy, involves a non-invasive procedure for taking certain tumor products from the blood, making it possible to extract as much diagnostic and predictive information as possible. Because of their close correlation with overall survival and progression-free survival, CTCs promise to become one of the leading tumor markers in the near future. In addition, their early presence in the blood can be used to detect solid tumors, provided that the problem of low sensitivity is solved.

The detection of CTCs is however challenging because their number can be very small and not uniformly distributed into the bloodstream. The CTC detection is difficult, however, as their numbers can be very small and they are not evenly distributed in the blood. CTCs can have an average density of less than one cell per ml, up to more than 10 cells per ml of blood in patients with metastatic cancer, while white blood cells are in the millions and globules red in the billion [1].

There are several CTC detection systems, but the only one approved by the Food and Drug Administration (FDA) is CellSearch (Veridex) [2]. Although it is considered the reference in this field, this method of semi-automated immunomagnetic isolation based on anti-EpCAM antibodies does not identify any CTC in a third of patients with breast cancer [3] or metastatic prostate cancer [4]. This may be due to the fact that CTCs were not present in the blood samples (the device uses blood samples of only 7.5 ml), or to the fact that they are present but they are not detected in because of low expression of EpCAM on their surface (antibody bias). In addition, the device requires the intervention of a qualified human operator to verify and select the correct CTC images from those of the camera, which makes the process tedious and expensive.

Other methods designed for CTC detection are presented in [5–10]. An interesting review is presented in [5], where CTC detection and isolation methods are classified according to their detection principle: nucleic acid-based methods, physical-based methods (based on size and mechanical plasticity), methods based on specific antibodies (immunocytochemistry, immunomagnetic and adhesion-based methods). Current detection methods have certain limitations: the antibody-based methods are biased by the characteristics of the antibodies used or by the variable expression of the corresponding antigens, whereas the methods based on the physical properties of the CTCs are biased by the heterogeneity of size or their mechanical properties. For example, in some prostate cancers, CTCs may have sizes similar to nucleated blood cells, making it difficult to apply a size-based detection filter. However, oncology practice requires a sensitive detection method to monitor CTC over long periods of time to compensate for extreme scarcity in the blood. In addition, the analysis must be repeated several times, if necessary, to monitor response to therapy and provide personalized treatment.

To overcome some of these limitations, we propose another method of identifying CTCs in the blood. This consists of identifying CTCs in unstained blood microscopic images, based on image features analysis and boosting techniques. Since CTCs are malignant transformed cells, their morphology differs from that of normal blood cells: larger size, irregular contour, atypical nucleus (higher density, greater density, nonhomogeneous chromatin, irregular contour, thicker nuclear membrane), increased nucleo-cytoplasmic ratio. These indicators of malignancy are used in the routine work of cytologists and pathologists to diagnose cancer using a microscope. We assume that this process of visual cell analysis can be automated. By exploiting the morphological differences described above, “imagistic features” have been proposed to model each type of cell.

This work is meant to be the first step in a future project to identify and isolate CTCs from the blood of cancer patients in real time, by continuously processing large amounts of blood that will be reused just as for dialysis. Such a system requires the use of (i) unstained blood samples, (ii) live cell imaging techniques, and (iii) a powerful CTC detection technique that can differentiate them from normal blood cells. Some of the most popular label-free microscopic techniques used for living cells are phase contrast (PhC), differential interference contrast (DIC), dark field (DF), and even digital holographic microscopy. For our study, we chose the DF technique for its simplicity and the very good quality of live cell images, with many details of the cellular structure. These make it easy to differentiate CTCs from other blood cells.

To date, to our knowledge, there are few studies on the morphological identification of CTCs in unstained blood. Some articles deal with the question of the classification of normal blood cells. Such an example is presented in [11] where the authors attempted to create a new portable analyzer for hematology. They used a lens-free holographic microscope to visualize blood cells passing through a microfluidic chip and to classify leukocytes into three types of cells: granulocytes, monocytes, and lymphocytes, with a sensitivity greater than 85%. Most studies on unstained specimens focus on the monitoring of cell cultures over time, in order to observe and understand their spatial behavior over time. Some of the studies on monitoring cell populations are listed below [12–14].

In this work, we propose a new approach for the live detection of CTCs. For this, we generated a new set of DF microscopic image data containing three types of cells spiked in non-stained normal blood; we designed new booster classifiers for detecting these cells in DF images using a convolutional approach and conducted a performance study on CTC detection algorithms, comparing them to fluorescence images—considered the gold standard—as well as to the results obtained by human experts.

## Method

This section discusses aspects of tumor cell culture, the spiking of tumor cells in blood from healthy donors, and the preparation of microscopic specimens for the detection of tumor cells. We also present here our proposed method for detecting tumor cells in native blood samples based on image processing and machine learning techniques, as well as validation methods. The protocol of the study was approved by the Ethics Committee for Clinical Research of the University of Medicine and Pharmacy “Iuliu Hatieganu” Cluj-Napoca, Romania.

### Cell cultures

We used two breast cancer cell lines, Hs 578T and MCF7, as well as the DLD-1 (CCL-221) cell line derived from colorectal adenocarcinoma. The cells were cultured according to the suppliers instructions (European Collection of Authenticated Cell Cultures- ECACC). After culture, cells were harvested using 0.25% trypsin + EDTA. We used only cell suspensions with a viability of over 90%, as assessed by trypan blue test. All cell culture reagents were purchased from Sigma-Aldrich (St. Louis, MO, USA).

### Cell spiking and preparation of microscopic samples

The cells were spiked in 1 ml of peripheral blood, collected on EDTA, from healthy donors. We will call these tumor cells later as “CTCs”. The concentration of spiked cells was adjusted to 500,000 cells / ml of blood. Native cytological preparations (unfixed and unstained) were made from spiked blood using standard histological glass slides (BRAND microscope slide, Sigma-Aldrich) and 20 × 20 mm pre-cleaned cover glasses with a thickness of 0.13 to 0.17 mm made of borosilicate glass (BRAND, from Sigma-Aldrich). The distance between the slide and the cover glass was fixed at about 7 μm using polystyrene microbeads as spacers (78462 SIGMA microparticles based on polystyrene, Sigma-Aldrich). For each cell line, 3 to 5 different slides were used and processed on different days. Virtually all microscopic fields containing at least one tumor cell were evaluated on each slide included in the experiment, with the exception of those containing significant artifacts that would have affected the evaluation.

### Microscope setting and image acquisition

To visualize our native (unstained) samples, we chose the dark field microscopy (DF) technique, which provides a good quality of microscopic images, with minute details of the intracellular structure. The images were acquired using a XIMEA XiQ color camera (MQ022CG-CM) mounted on an Olympus BX43 fluorescence microscope, equipped with a cardioid condenser for DF illumination, with an 20x objective. In order to correctly identify, without any doubt, the presence of viable tumor cells (CTC substitutes) in microscopic samples, each image was photographed in duplicate, one in fluorescence and the other in DF. Only images taken in DF format have been used in the image analysis process. Fluorescence images were used only as a “golden standard” to verify the accuracy of cell classification by computer software.

### CTC detection method

The proposed detection method uses a convolutional approach inspired by the concepts of Integral Channel Features [15] and Convolutional Neural Networks (CNN). A similar approach was previously used in [16, 17] for the classification of multi-class objects in traffic scenes, thus obtaining real-time performance and very good sensitivity. In [18], we proposed a method of classifying CTCs using several image characteristics, such as histogram statistics, grayscale co-occurrence matrix, gray tone difference matrix, color staistics and some new radial features, a method validated by a high classification rate. Motivated by our previous results and the computational advantages of a convolution approach, we propose in this paper a CTC detection method using a series of filters that focus on the image characteristics validated in [18]. The current method uses the Adaboost classifier for training and CTC forecasting.

The characteristics are calculated at three different image resolutions in order to capture both the fine details of the tumor cell morphological characteristics as well as the rougher ones, acting as the average pooling layers in a CNN.

The values of the feature vector are those of the coordinates of the current pixels in all the convolution channels and also those of several other neighboring pixels. We define this neighborhood as the set of pixels covering the area of the tumor cell. In doing so, we are able to provide a representation that models the texture of the cell.

**Fig 1:**
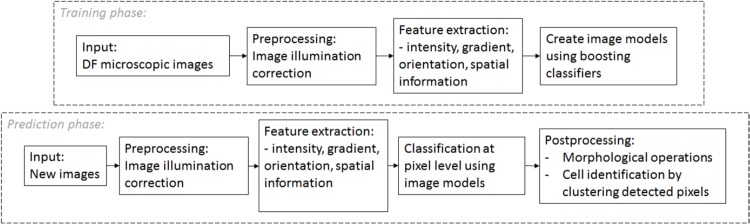
Block diagram of the proposed method.

The block diagram of the proposed approach is shown in Fig 1.

Like any classification problem, our method has two main phases: training and prediction. In each case, we begin with a preprocessing step to increase image quality by reducing image artifacts. Next, we apply convolution filters that focus on features such as: color, image gradient, gradient orientation, and spatial information. During the learning phase, we construct classifiers capable of discriminating pixels belonging to the tumor cells of pixels belonging to healthy blood cells or in the background. In the prediction phase, we classify all the pixels of the test-images in CTC or non-CTC pixels. And finally, we obtain the semantic segmentation of the DF image after some post-processing operations:

1. apply morphological operators to eliminate small groups of detected pixels
2. and label the connected detected pixel groups. The detected objects are validated as CTCs if their area is greater than a certain value (in our experiments, this threshold value is set to 100).

In the next subsection, we will detail each of these steps.

### Illumination correction in DF microscopic images

**Fig 2:**
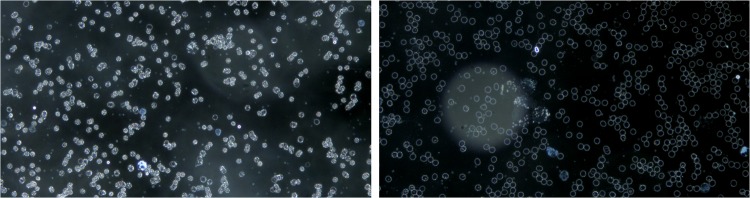
Variation of illumination of DF microscopic images due to artifacts.

**Fig 3:**
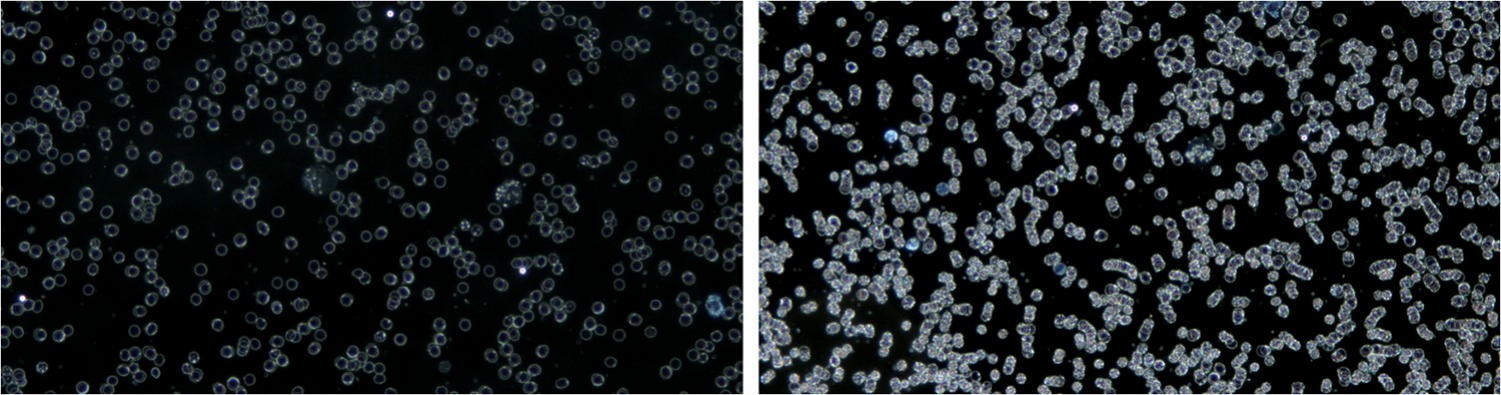
Variation of illumination in DF microscopic images due to the density of different cells in the image.

There are several sources of illumination variability for DF images. The main ones are:

- the artifacts on the cover glass above the blood sample are highly reflective in DF microscopy and, since they are not usually in the focal plane of the cells, they appear in the form of bright spotted halos (see Fig 2),
- the different cell density in the image influences the overall brightness of the image (see Fig 3).

**Fig 4:**
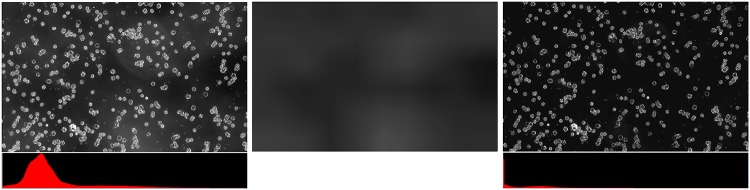
Illumination correction: Original image (left), estimated noise (middle) and the corrected image (right).

These variations can alter the results of the classification because all the entities are calculated according to intensity levels. In order to overcome this problem, we propose a lighting correction method acting as a pre-treatment step. According to the image acquisition principle in DF, in which the background of the image is black, we expect the highest bin of the histogram of the image to be black. Generally, this rule is confirmed except for images corrupted by an artifact, where the histogram with the highest bin is moved to the right (see Fig 4). As a result, the proposed method is to search for the background bin that corresponds to the highest bin of the histogram and to shift the entire histogram in the left direction to establish the value of the histogram. background to zero (black). Since artefact-induced scatter light is not constant on the image surface, this process is performed for the *wsize* × *wsize* subpictures and recombines the results by linear interpolation.

Let *I*_*src*_ be the source image (see Fig 4, left image), *h*_*src*(*i*,*j*)_ the histogram of a *wsize* × *wsize* subimage with the high left corner at position (*i*, *j*) and *g*_*max*(*i*,*j*)_ the intensity level corresponding to the highest bin in *h*_*src*(*i*,*j*)_. The *g*_*max*_ values are combined trough linear interpolation to form the artifact image mask *I*_*mask*_ (see Fig 4, middle image) of same size as *I*_*src*_. Then, the illumination correction is the defined as:

$$\begin{array}{c} I_{dst}\left(i,j\right)=I_{src}\left(i,j\right)-I_{mask}\left(i,j\right) \end{array}$$ (1)

where *I*_*dst*_ is the corrected image (see Fig 4, right image), and (*i*, *j*) ar the image coordinates.

### Feature extraction

Using the terminology of [15], given *I*, an input image, its corresponding channel is an input image recorded map, where the output pixels are calculated from the corresponding input pixel patches. A trivial channel is the grayscale image, while each color image is a channel. The channels used in our work are:

- the first three contain information about colors in the CieLuv color space, because of their applicability to colored lighting. The CieLuv color space provides a perceptually uniform color space in which the distance between two points roughly indicates how the colors are different in luminance, chrominance and hue [19].
- the next two channels contain the magnitude and orientation of the gradient. The orientation of the gradient is an integer between {0, 1, 8…, 7} which codifies the corresponding angles around {0°, 45°, 90°, 135°, 180°, 225°, 270°, 315°}.
- the following channels contain the same convolutions applied to the subsampled images of dimensions (*height*/2, *width*/2) and (*height*/4, *width*/4) respectively.

**Fig 5:**
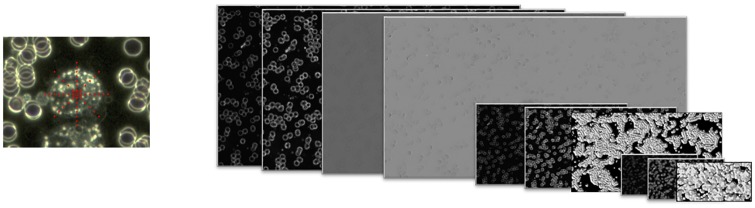
Extraction of characteristics: Location of neighboring pixels in red (on the left); multi-resolution convolution channels (right).

Fig 5 shows in the left-hand sub-figure the coordinates of the neighboring pixels used to characterize the central pixel and provides a visual representation of the convolution channels in the right.

### Classification

For classification, we use the Adaboost classifier [20] to create imagistic models of CTC cells based on the convolution characteristics described in the subsection above and to classify the pixels of the image as CTC or nonCTC.

### Validation

The validation of our detection method is carried out in two different ways: by comparison with the fluorescently labeled cells used as gold standard and by comparison with the results obtained by the human expert. All comparisons were made using several measures resulting from a confusion matrix, such as sensitivity (TP rate) and estimated specificity (TN rate).

To identify viable tumor cells with maximum certainty, they were fluorescently labeled. The Hs 578T, MCF7 and DLD-1 cells were fluorescently labeled with the PKH26 membrane linker, which is also an indicator of viability. A PKH26 kit (Sigma-Aldrich) was used; this membrane dye does not influence cell viability and retains their fluorescence for a long time. The tumor cells in suspension were centrifuged at 1000 rpm for 5 minutes and the cell pellet was washed a further 2 times with PBS. The cells were then counted and resuspended in the kit diluent (106 cells / ml) and then 1 ml of diluted PKH26 dye (4 μl / ml diluent) was added. Cells were exposed to dye for 5 min and staining was stopped by addition of 10 ml of complete RPMI culture medium supplemented with 10% fetal serum. Two further washes were performed with 10 ml of the same complete RPMI medium supplemented with 10% FCS. The fluorescence of the cells was monitored with a Zeiss Axiovert D1 inverted fluorescence microscope, using a 546 nm filter. Each CTC detected by the proposed method was then validated by comparison with fluorescence images.

For the second method, we used two experts in citologic and anatomo-pathologic diagnosis. They were asked to independently mark the CTCs in all the images in our dataset. After prior validation with the fluorescent images, their results were compared with the results of the proposed method.

## Results

### Dataset

**Fig 6:**
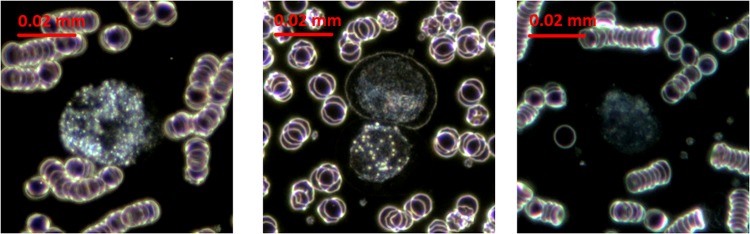
Different types of CTCs: HS 578T (left), MCF7 (middle), DLD-1 (right).

Our dataset contains 263 DF images summarizing a total number of 632 CTC cells, belonging to the three cell lines used in this study, as follows: HS 578T (83 images, 235 CTCs), MCF7 (60 images, 200 CTCs) and DLD (120 images, 197 CTCs), as shown in Fig 6. The average diameter of tumors cells is as follows: Hs 578T = 23.15*μm*, MCF7 = 21.75*μm*, DLD-1 = 18.13*μm*.

**Fig 7:**
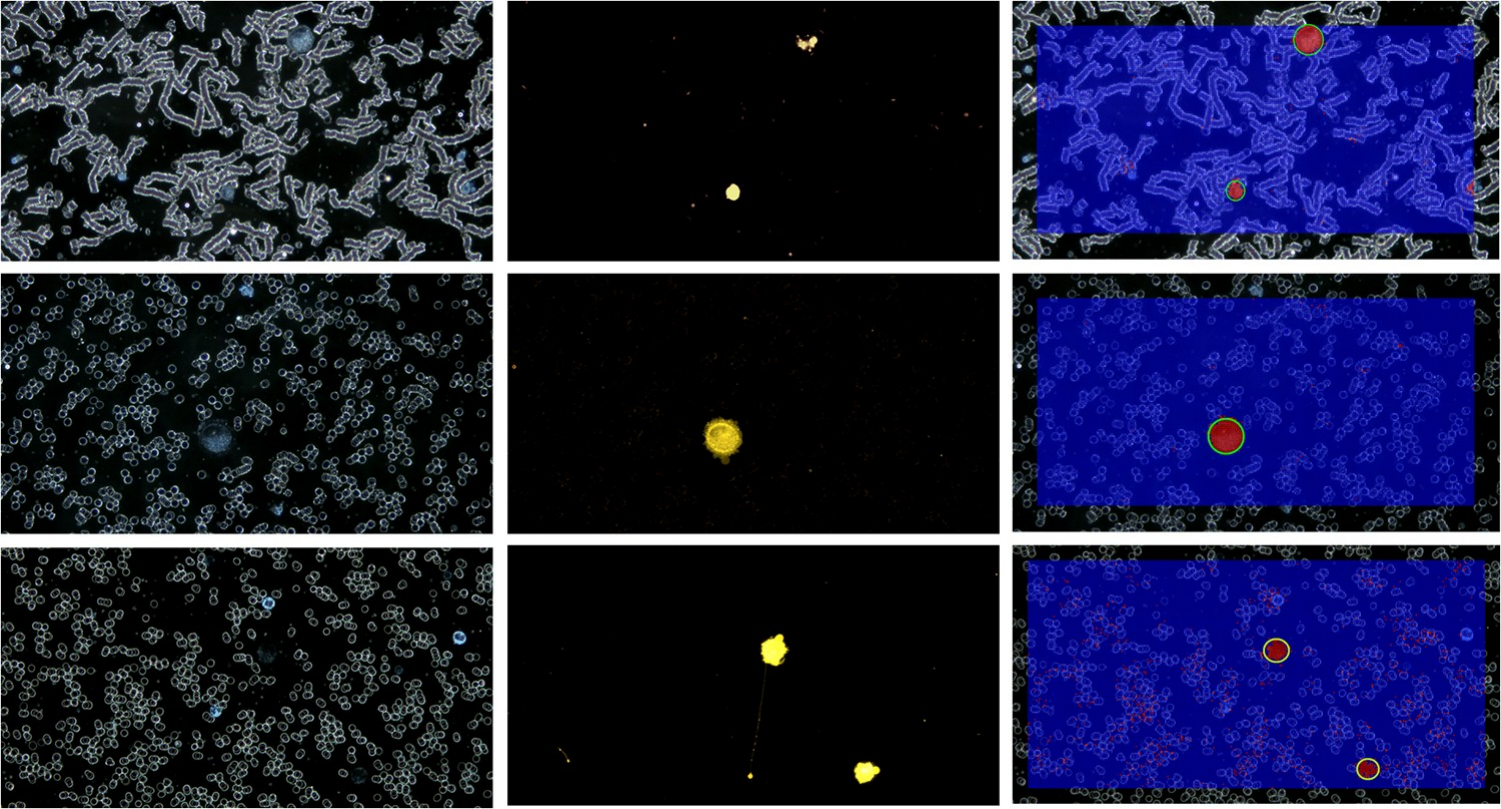
Qualitative classification results: Original images in DF(left); fluorescent image (middle); detection results (right). Each line corresponds to a different dataset: HS 578T, MCF7 and DLD-1 (from top to bottom).

In parallel with DF images, our data set contains paired fluorescence images used as “Ground Truth” (GT) in our experiments. A visual example of such pairs of images is shown in Fig 7, in the first two columns.

### Classification results

Four models were built: one for each dataset (HS 578T, MCF7, DLD-1) and a combined one (HS 578T+MCF7+DLD-1, further referred as “Generic Classif.”) trained on samples from all datasets. For classification testing and validation we used 10 fold cross validation and we use validation metrics such as: TP (true positive) indicating the number of CTCs that were correctly classified, TN (true negative)—the number of CTC that were not detected and FP (false positive)—the number of other cells/ or group of cells that were detected as CTCs. Sensitivity is measured as TP rate. Other metrics that requires TN (true positive) rate cannot be computed because we do not have the actual number of the other cells present in the images. Still, these values can be estimated by taking into account an average number of cells per image. We obtained an average value of 1270 cells per image obtained through a sampling process performed on all the images in our dataset. Consequently, we can also infer estimative values for accuracy and specificity.

The validation was performed in comparison with the images in fluorescence—considered the ground truth as well as in comparison with human expert.

#### Comparing results with fluorescence images

For each image in the testing set, the classification was performed at pixel level.

Some qualitative results of this classification are shown in Fig 7. The images in each line of this figure correspond to different dataset: HS 578T, MCF7 and DLD-1 (from top to bottom). In the columns are depicted from left to right original images in DF, fluorescence images and the labeled images resulted from the automatic detection process. Red pixels in the result images belong to *CTC class* and blue pixels to *Other class*. After morphological operations and object labeling, the validated CTCs are marked with green circles. An example of false positive is shown in 3^rd^ line, 3^rd^ column.

Quantitative results are shown in Tables 1 and 2. In Table 1, the results were obtained on samples from the same category as the ones used for training (e.g. HS 578T model was tested on HS 578T images and so on). The TP, TN and FP rates are shown for each testing case.

**Table 1:** Quantitative evaluation of the four classification models.

Training dataset	Testing dataset	# Images	# CTCs	TP	TP rate (sensitivity)	FN	FN rate	FP	Estimated FP rate
HS 578T	HS 578T	83	235	230	97.87%	5	2.13%	1	0.0009
MCF7	MCF7	60	200	185	92.50%	15	7.50%	14	0.0183
DLD-1	DLD-1	120	197	168	85.27%	29	14.72%	36	0.0236
Combined dataset	Combined dataset	263	632	587	92.87%	45	7.12%	51	0.0142

**Table 2:** Quantitative evaluation of the general classification models (Generic Classif.) applied on individual datasets.

Testing datasets	# Images	# CTCs	TP	TP rate (sensitivity)	FN	FN rate	FP	Estimated FP rate
HS 578T	83	235	229	97.44%	6	2.66%	5	0.0047
MCF7	60	200	190	95.00%	10	5.10%	52	0.0682
DLD-1	120	197	163	82.74%	34	17.25%	36	0.0236

In order to verify the robustness of the resulted Generic Classifier, this was tested on each of the individual cell line’s dataset. The results are shown in Table 2, proving the stability of the results.

#### Comparing results with human expert

**Fig 8:**
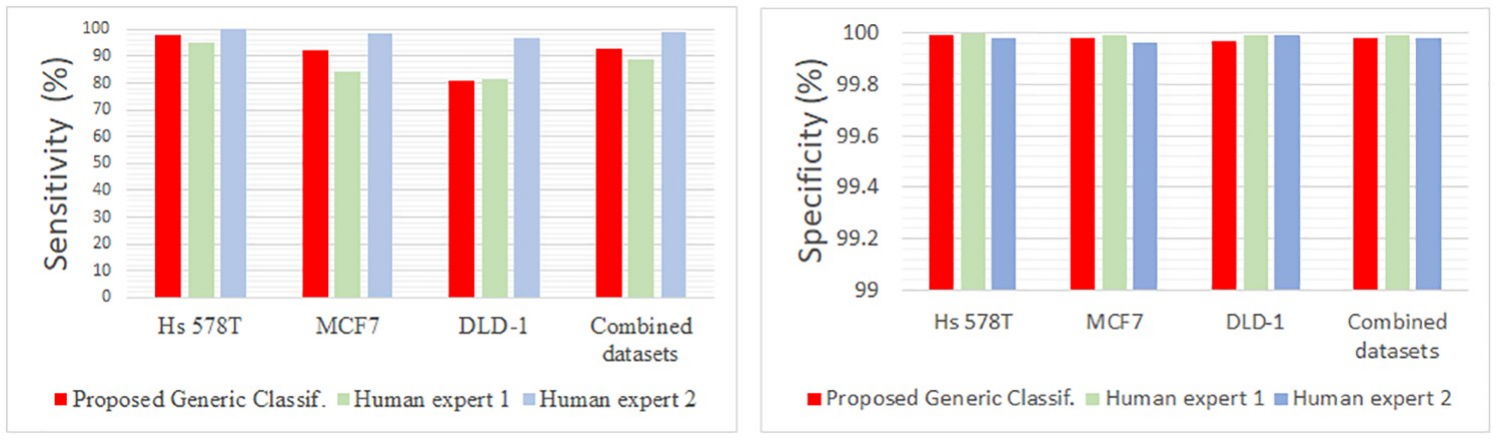
Comparing results with human expert—Visual charts.

The same statistics were computed for the results obtained by the human experts. The comparative results are shown in Table 3 and Fig 8.

**Table 3:** Comparing results with human expert.

Validation metric	Sensitivity (TP rate)				estimated Specificity (TN rate)			
Dataset	HS 578T	MCF7	DLD-1	combined datasets	HS 578T	MCF7	DLD-1	combined datasets
Generic Classif.	97.87%	92.50%	86.29%	92.87%	99.99%	99.98%	99.97%	99.98%
Human expert 1	95.32%	84.50%	81.37%	88.64%	100%	99.99%	99.99%	99.99%
Human expert 2	100%	98.50%	97.06%	98.88%	99.98%	99.96%	99.99%	99.98%

Sensitivity differences between the two human experts could be explained by their different working philosophies. First expert marked as CTC only those cells about which it was absolutely certain they were CTCs, while the second expert marked all cells susceptible of being CTCs. It can be noticed that there is a tradeoff between sensitivity and specificity, as a high sensitivity brings with it higher number of FPs.

The results obtained by the proposed method were very close to those obtained by human experts, as they seem to fall in between those of the two philosophies. The robustness of the proposed method should be noticed, as the performance obtained for each cell line is very close to the results obtained by the generic classifier trained on the consolidated image set. This demonstrates that the proposed algorithm is not sensitive to the type of CTCs, promising good results for other types of cancer cells too.

## Conclusion

In this paper we proposed a new label-free method for identifying the presence of tumor cells in the blood, based on specific morphological characteristics, through an automated machine learning process.

The developed algorithm manages to detect tumor cells with a sensitivity of 92.87% and a specificity of 99.98%, values that fall within the range of those obtained by human experts. The algorithm proved not to be significantly influenced by the type of the studied tumor cell.

This robustness, along with its scalability, promises to make it suitable for identifying other types of circulating tumor cells. However, in order to achieve this goal, new studies are needed on an extended number of tumor cell lines, as well as on clinical samples from cancer patients.

## Supporting information

S1 Dataset (ZIP)
