# Supplementary figures and images for: Automatic detection of circulating tumor cells in darkfield microscopic images of unstained blood using boosting techniques
Source: PLoS One. 2018 Dec 13;13(12):e0208385. doi: 10.1371/journal.pone.0208385 (PMC6292606; doi:10.1371/journal.pone.0208385)

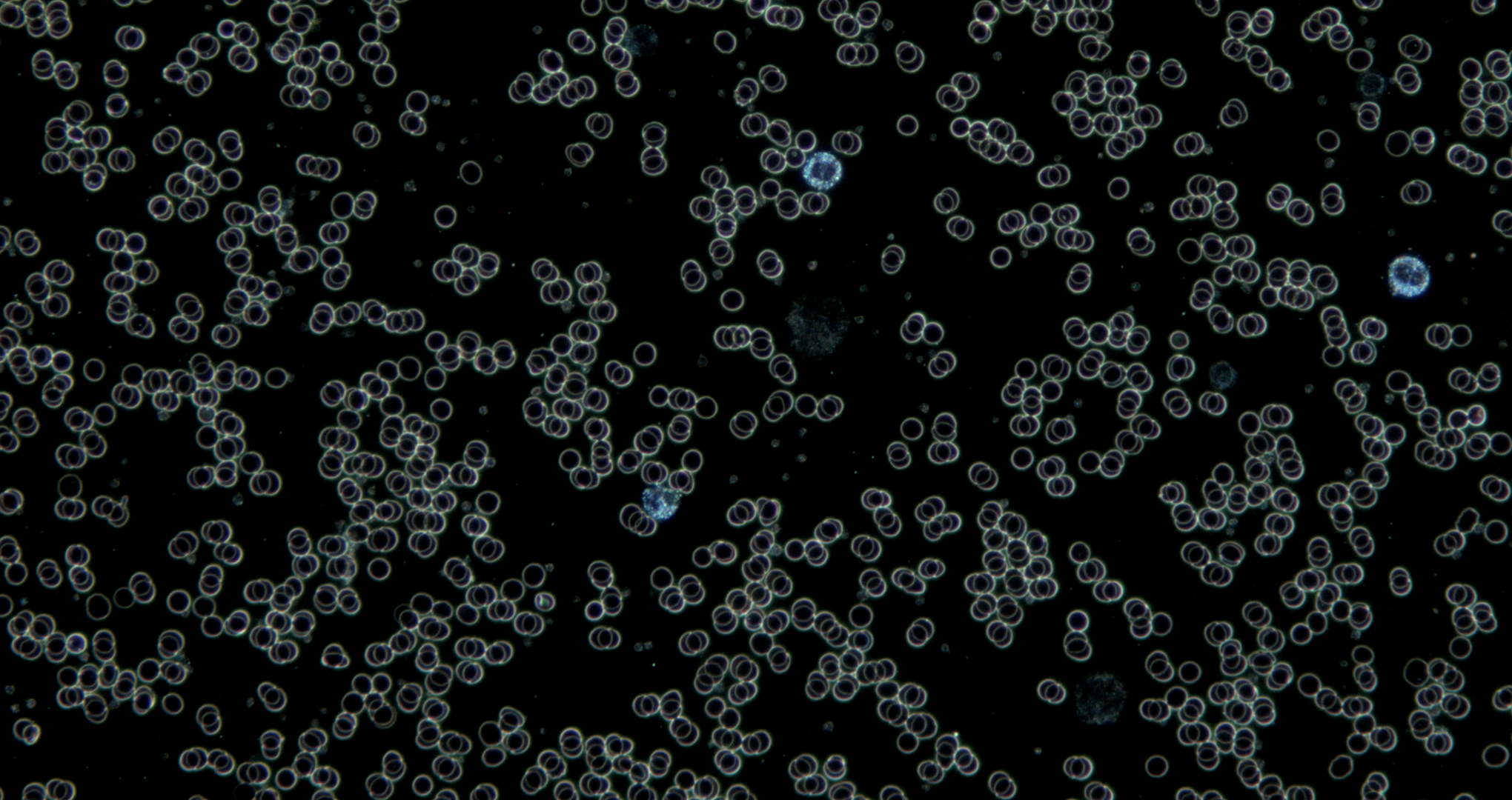

Supplement: S1 Dataset — (ZIP) [file pone.0208385.s001.zip › Dataset/DLD-1_DF.png]

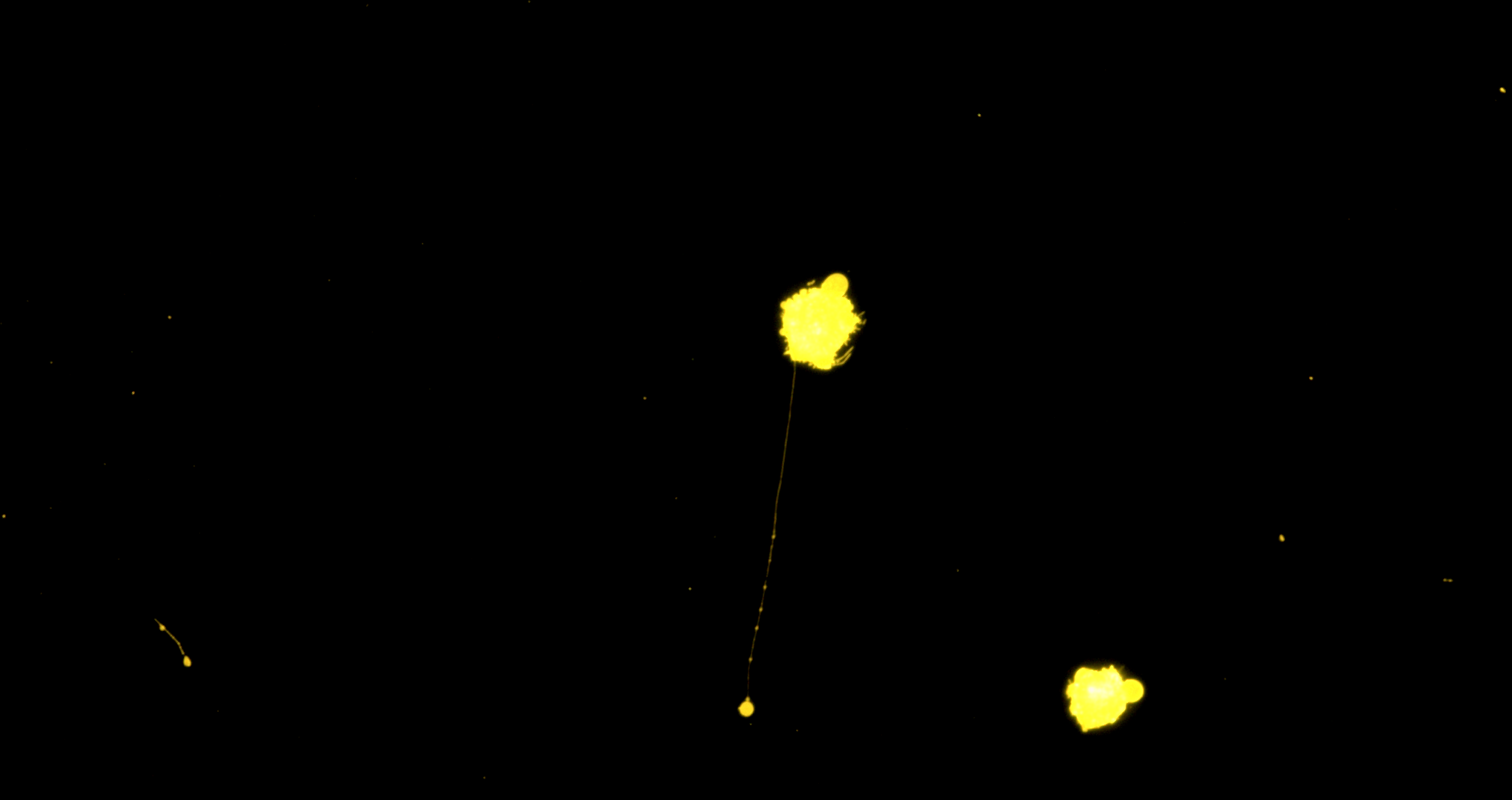

Supplement: S1 Dataset — (ZIP) [file pone.0208385.s001.zip › Dataset/DLD-1_fluorescence.png]

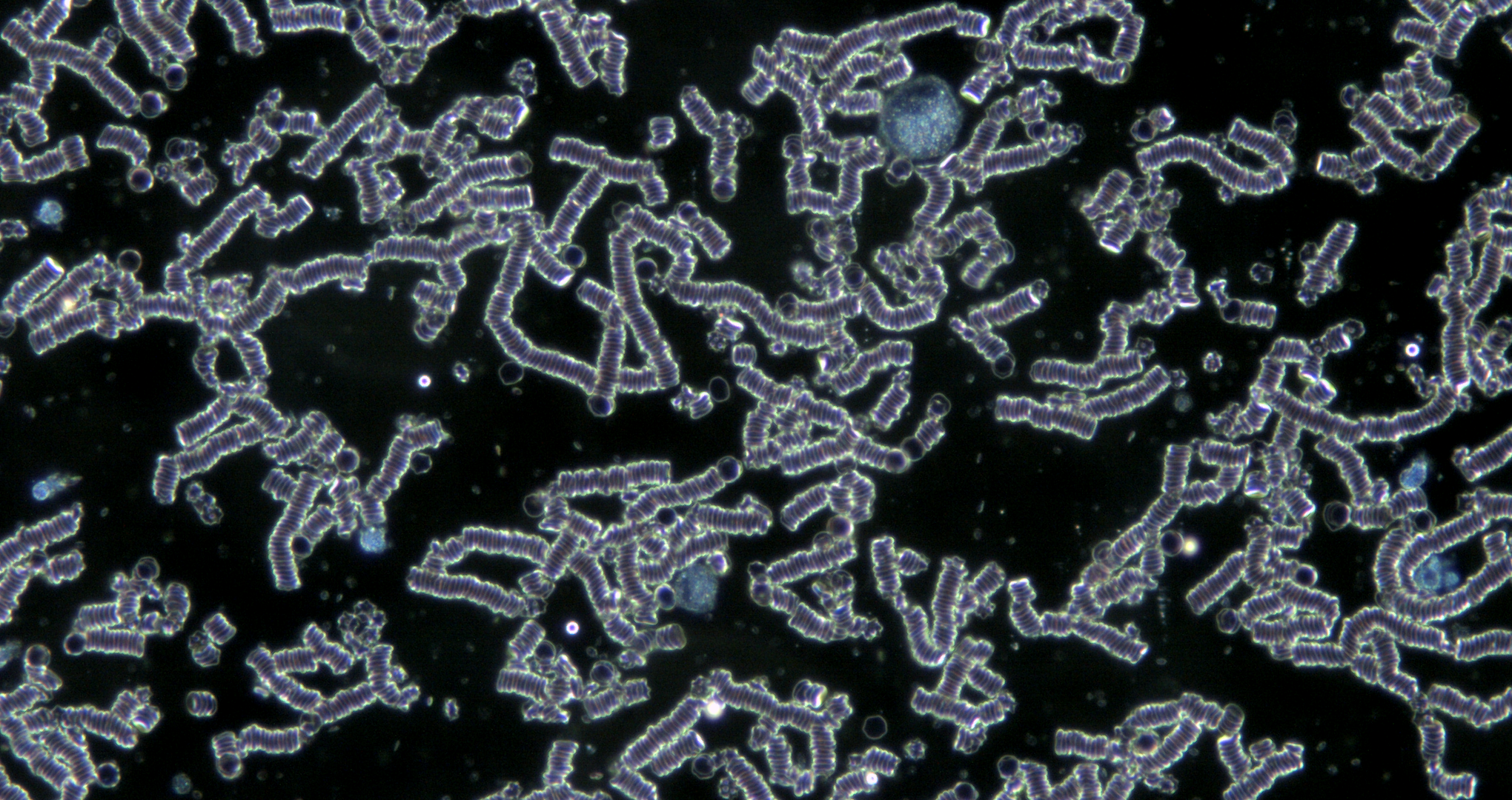

Supplement: S1 Dataset — (ZIP) [file pone.0208385.s001.zip › Dataset/Hs578T_DF.png]

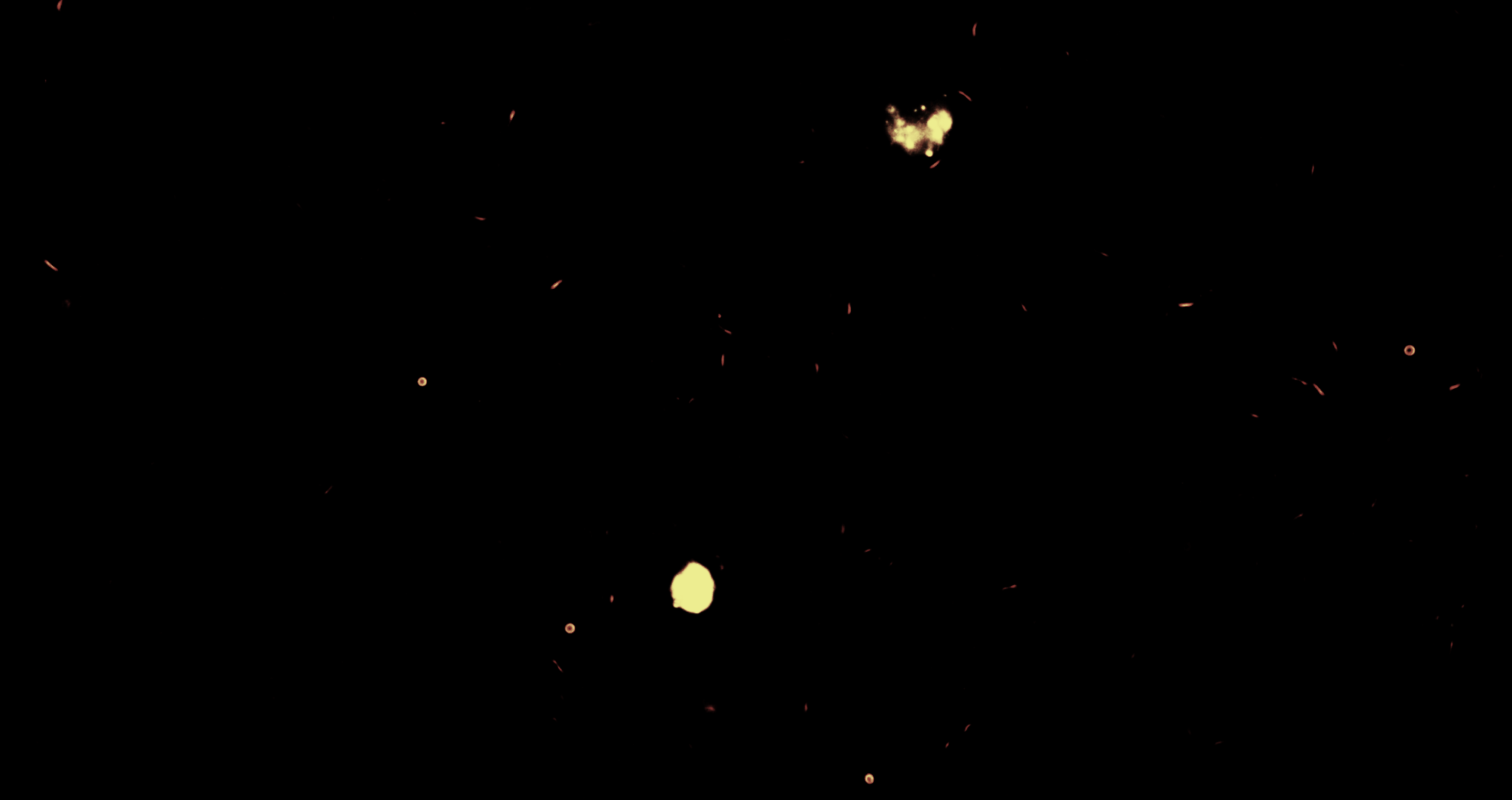

Supplement: S1 Dataset — (ZIP) [file pone.0208385.s001.zip › Dataset/Hs578T_fluorescence.png]

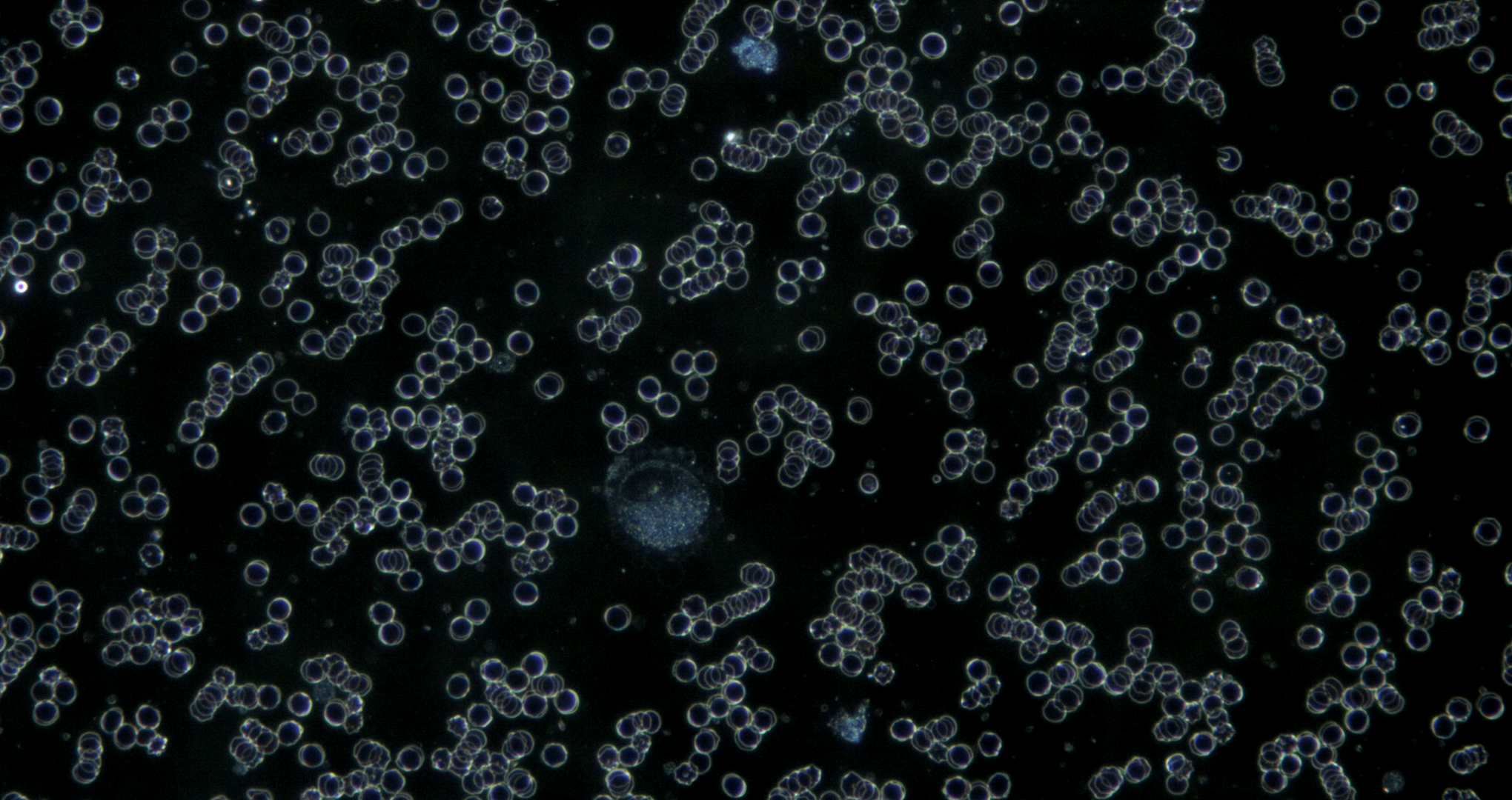

Supplement: S1 Dataset — (ZIP) [file pone.0208385.s001.zip › Dataset/MCF-7_DF.png]

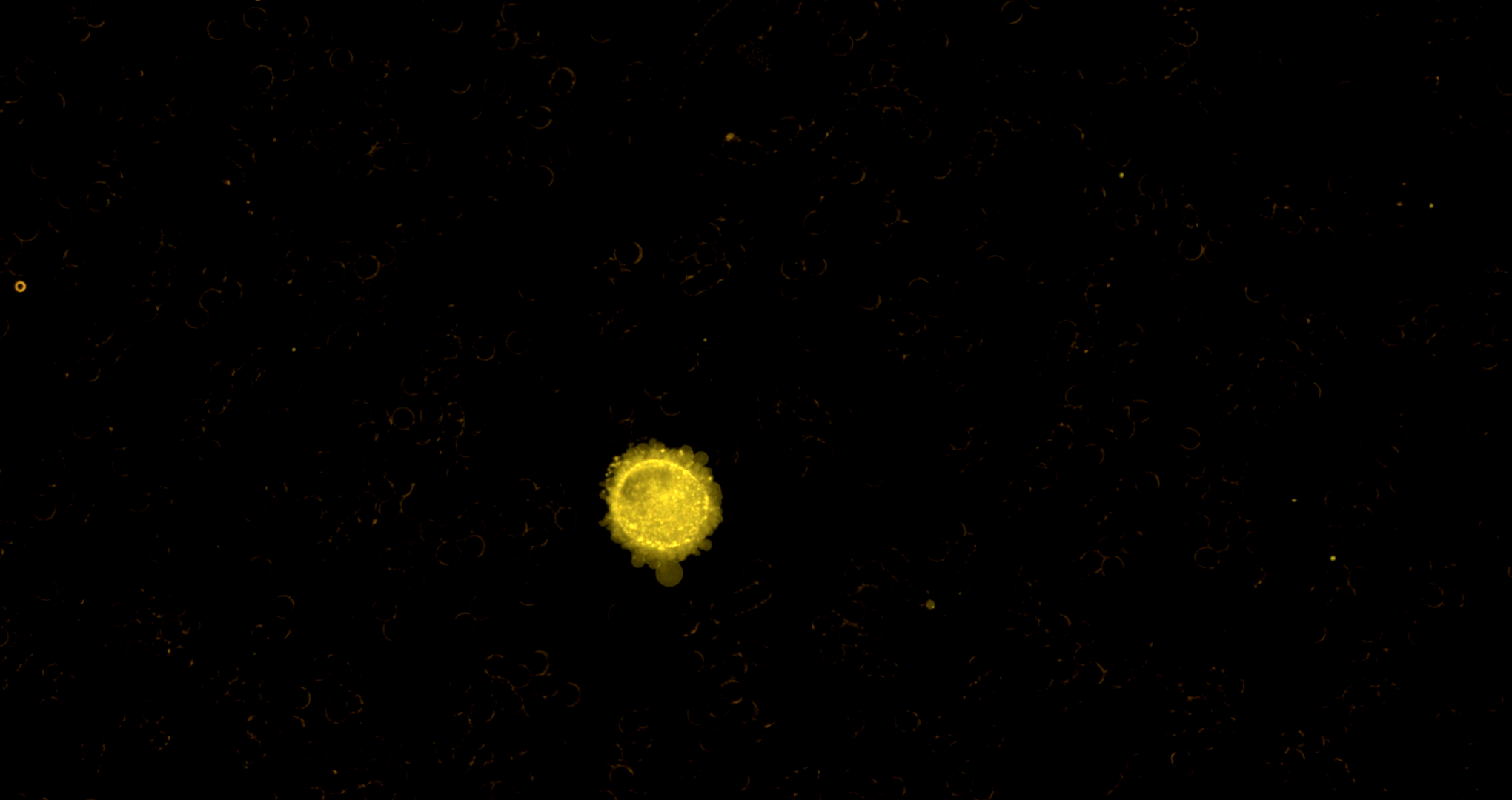

Supplement: S1 Dataset — (ZIP) [file pone.0208385.s001.zip › Dataset/MCF-7_fluorescence.png]
